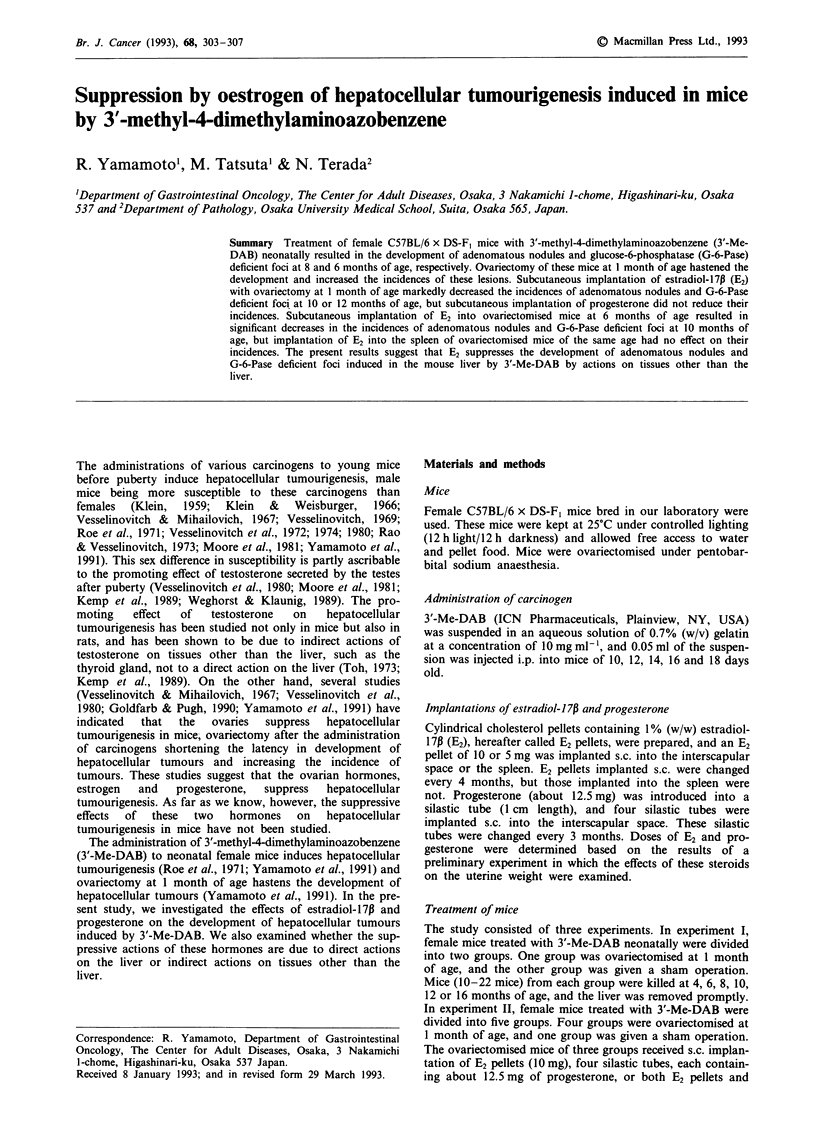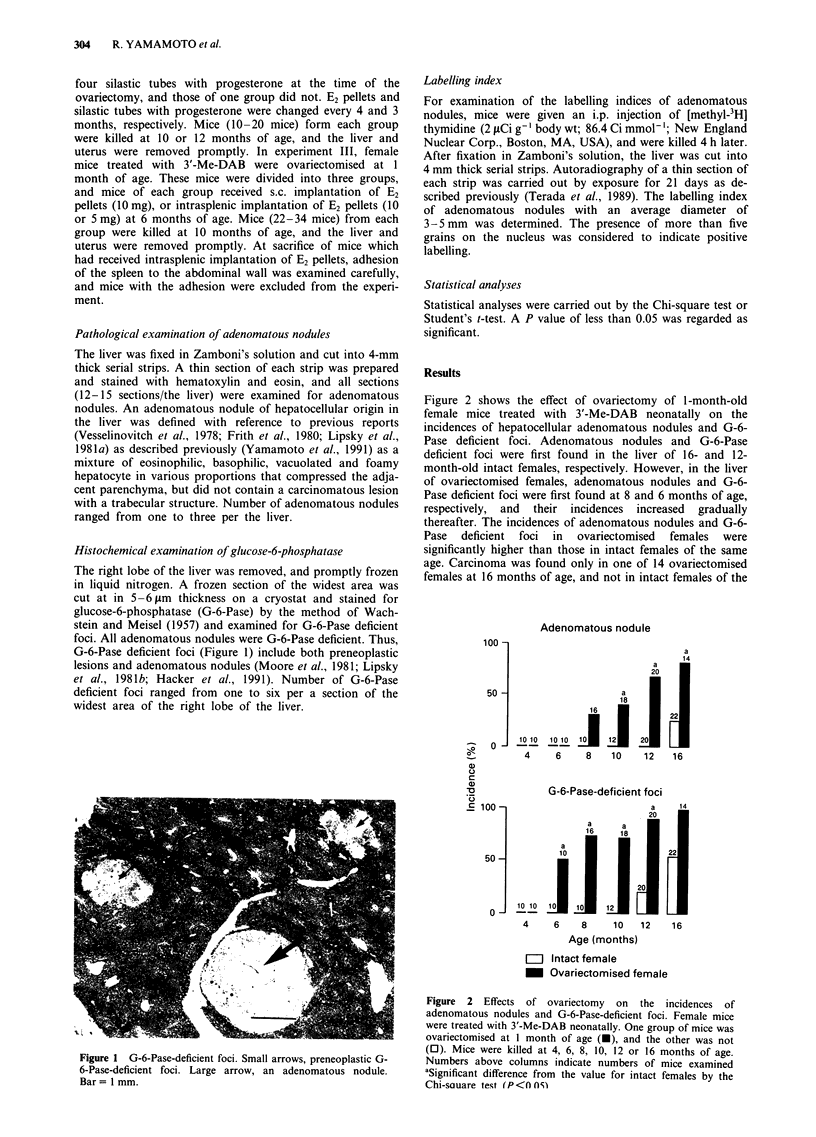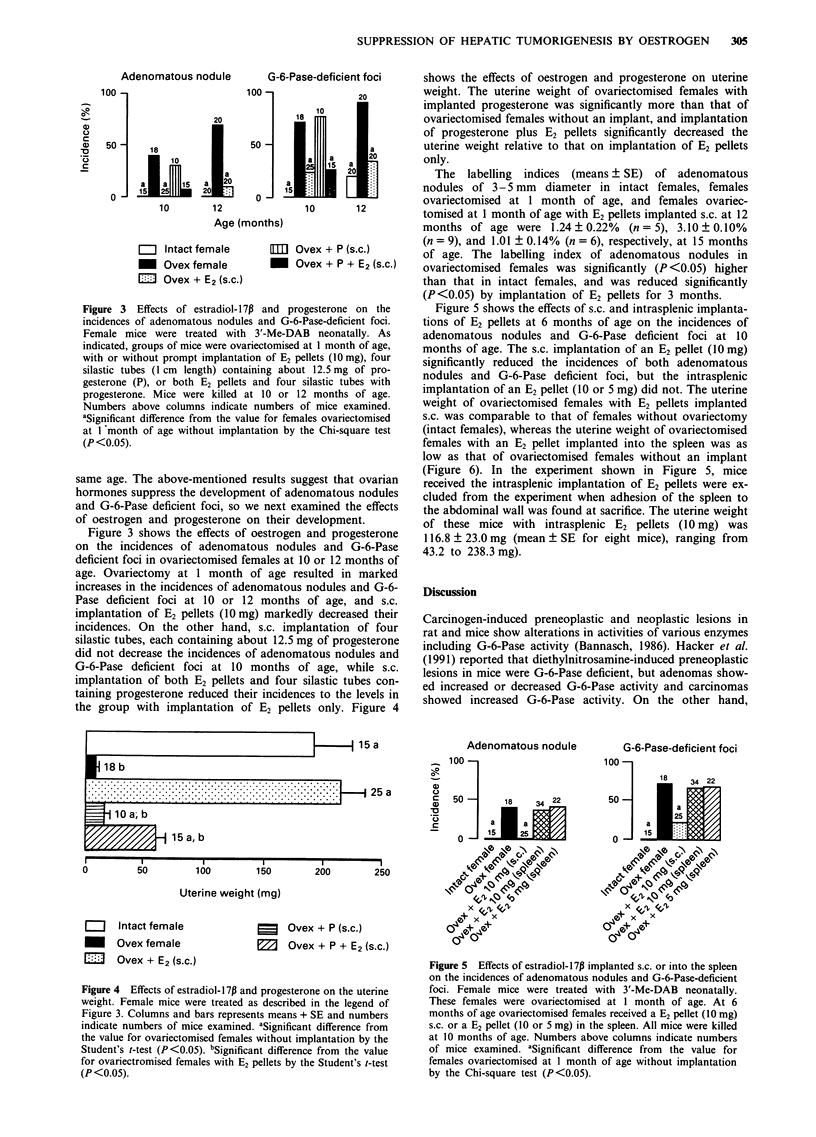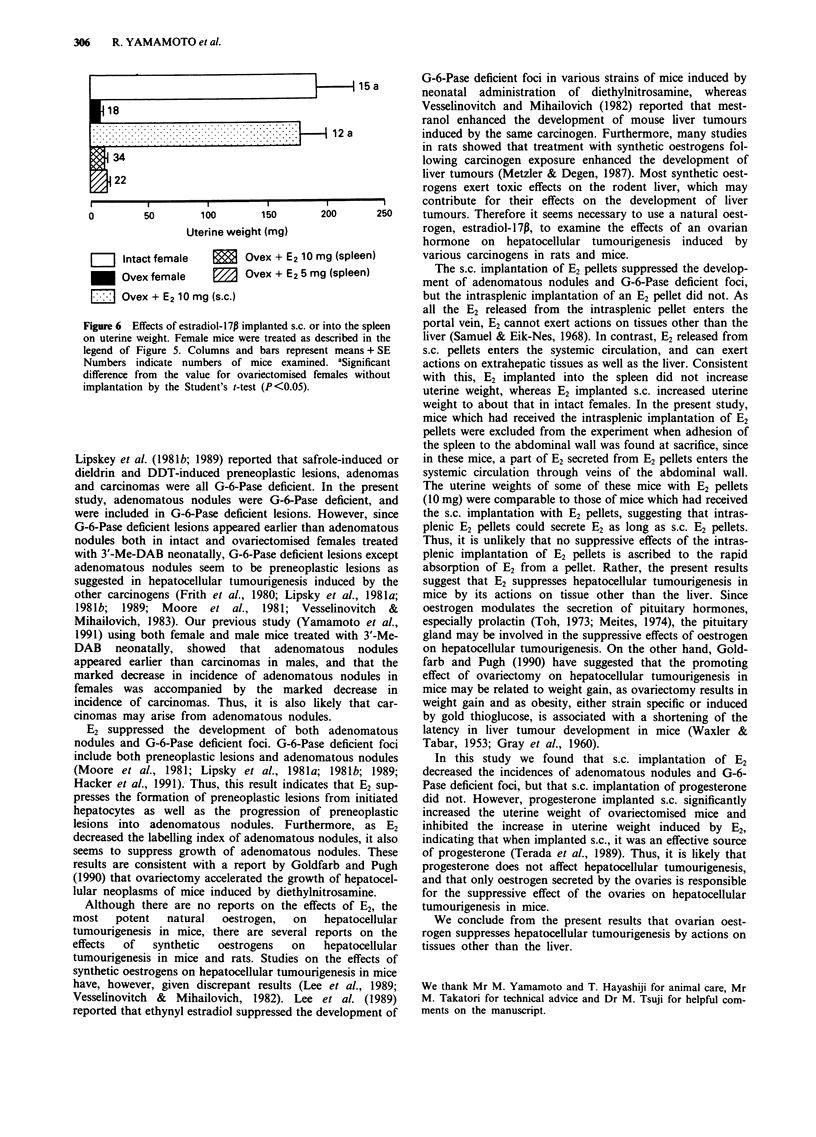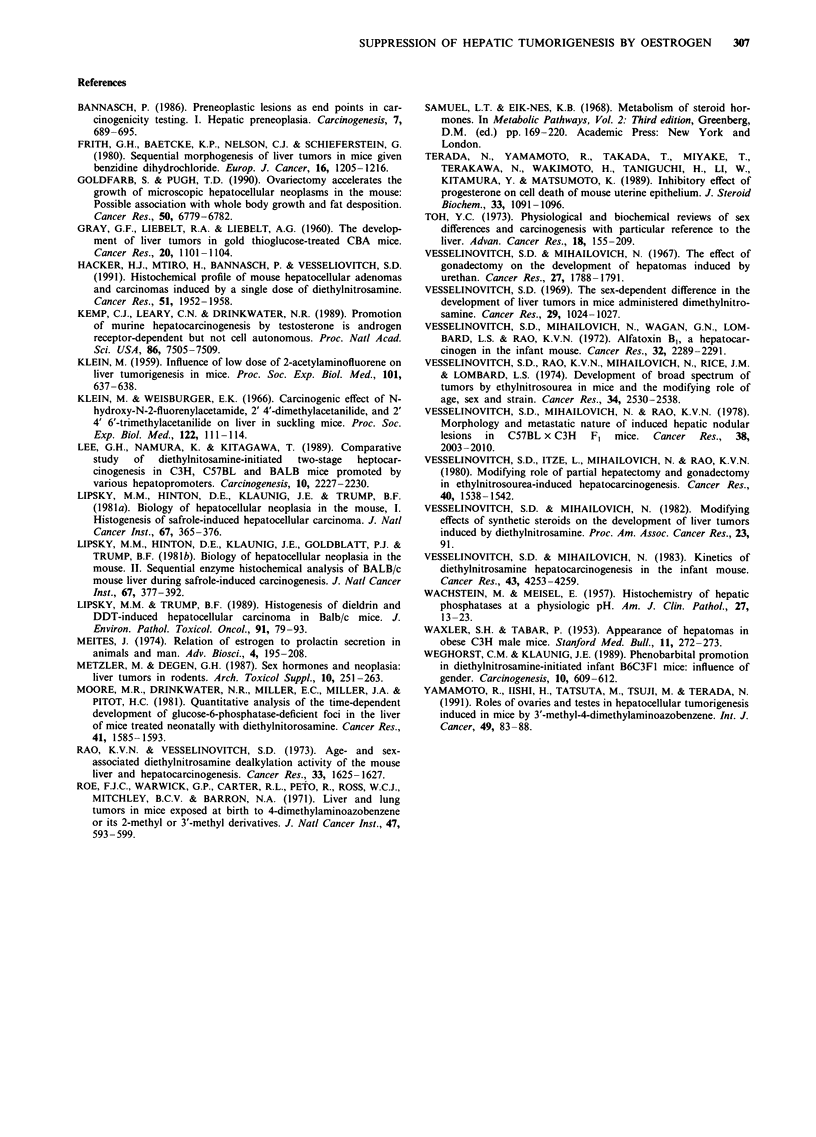# Suppression by oestrogen of hepatocellular tumourigenesis induced in mice by 3'-methyl-4-dimethylaminoazobenzene.

**DOI:** 10.1038/bjc.1993.332

**Published:** 1993-08

**Authors:** R. Yamamoto, M. Tatsuta, N. Terada

**Affiliations:** Department of Gastrointestinal Oncology, Center for Adult Diseases, Osaka, Japan.

## Abstract

**Images:**


Br. J. Cancer (1993), 68, 303-307 © Macmillan Press Ltd., 1993

### Suppression by oestrogen of hepatocellular tumourigenesis induced in mice by 3′-methyl-4-dimethylaminoazobenzene

R. Yamamoto[1], M. Tatsuta[1] & N. Terada[2]

[1]*Department of Gastrointestinal Oncology, The Center for Adult Diseases, Osaka, 3 Nakamichi 1-chome, Higashinari-ku, Osaka 537 and* [2]*Department of Pathology, Osaka University Medical School, Suita, Osaka 565, Japan.*

**Summary** Treatment of female C57BL/6 × DS-$F_1$ mice with 3′-methyl-4-dimethylaminoazobenzene (3′-Me-DAB) neonatally resulted in the development of adenomatous nodules and glucose-6-phosphatase (G-6-Pase) deficient foci at 8 and 6 months of age, respectively. Ovariectomy of these mice at 1 month of age hastened the development and increased the incidences of these lesions. Subcutaneous implantation of estradiol-17β ($E_2$) with ovariectomy at 1 month of age markedly decreased the incidences of adenomatous nodules and G-6-Pase deficient foci at 10 or 12 months of age, but subcutaneous implantation of progesterone did not reduce their incidences. Subcutaneous implantation of $E_2$ into ovariectomised mice at 6 months of age resulted in significant decreases in the incidences of adenomatous nodules and G-6-Pase deficient foci at 10 months of age, but implantation of $E_2$ into the spleen of ovariectomised mice of the same age had no effect on their incidences. The present results suggest that $E_2$ suppresses the development of adenomatous nodules and G-6-Pase deficient foci induced in the mouse liver by 3′-Me-DAB by actions on tissues other than the liver.

The administrations of various carcinogens to young mice before puberty induce hepatocellular tumourigenesis, male mice being more susceptible to these carcinogens than females (Klein, 1959; Klein & Weisburger, 1966; Vesselinovitch & Mihailovich, 1967; Vesselinovitch, 1969; Roe *et al.*, 1971; Vesselinovitch *et al.*, 1972; 1974; 1980; Rao & Vesselinovitch, 1973; Moore *et al.*, 1981; Yamamoto *et al.*, 1991). This sex difference in susceptibility is partly ascribable to the promoting effect of testosterone secreted by the testes after puberty (Vesselinovitch *et al.*, 1980; Moore *et al.*, 1981; Kemp *et al.*, 1989; Weghorst & Klaunig, 1989). The promoting effect of testosterone on hepatocellular tumourigenesis has been studied not only in mice but also in rats, and has been shown to be due to indirect actions of testosterone on tissues other than the liver, such as the thyroid gland, not to a direct action on the liver (Toh, 1973; Kemp *et al.*, 1989). On the other hand, several studies (Vesselinovitch & Mihailovich, 1967; Vesselinovitch *et al.*, 1980; Goldfarb & Pugh, 1990; Yamamoto *et al.*, 1991) have indicated that the ovaries suppress hepatocellular tumourigenesis in mice, ovariectomy after the administration of carcinogens shortening the latency in development of hepatocellular tumours and increasing the incidence of tumours. These studies suggest that the ovarian hormones, estrogen and progesterone, suppress hepatocellular tumourigenesis. As far as we know, however, the suppressive effects of these two hormones on hepatocellular tumourigenesis in mice have not been studied.

The administration of 3′-methyl-4-dimethylaminoazobenzene (3′-Me-DAB) to neonatal female mice induces hepatocellular tumourigenesis (Roe *et al.*, 1971; Yamamoto *et al.*, 1991) and ovariectomy at 1 month of age hastens the development of hepatocellular tumours (Yamamoto *et al.*, 1991). In the present study, we investigated the effects of estradiol-17β and progesterone on the development of hepatocellular tumours induced by 3′-Me-DAB. We also examined whether the suppressive actions of these hormones are due to direct actions on the liver or indirect actions on tissues other than the liver.

Correspondence: R. Yamamoto, Department of Gastrointestinal Oncology, The Center for Adult Diseases, Osaka, 3 Nakamichi 1-chome, Higashinari-ku, Osaka 537 Japan.
Received 8 January 1993; and in revised form 29 March 1993.

### Materials and methods

### *Mice*

Female C57BL/6 × DS-$F_1$ mice bred in our laboratory were used. These mice were kept at 25°C under controlled lighting (12 h light/12 h darkness) and allowed free access to water and pellet food. Mice were ovariectomised under pentobarbital sodium anaesthesia.

### *Administration of carcinogen*

3′-Me-DAB (ICN Pharmaceuticals, Plainview, NY, USA) was suspended in an aqueous solution of 0.7% (w/v) gelatin at a concentration of 10 mg $ml^{-1}$, and 0.05 ml of the suspension was injected i.p. into mice of 10, 12, 14, 16 and 18 days old.

### *Implantations of estradiol-17β and progesterone*

Cylindrical cholesterol pellets containing 1% (w/w) estradiol-17β ($E_2$), hereafter called $E_2$ pellets, were prepared, and an $E_2$ pellet of 10 or 5 mg was implanted s.c. into the interscapular space or the spleen. $E_2$ pellets implanted s.c. were changed every 4 months, but those implanted into the spleen were not. Progesterone (about 12.5 mg) was introduced into a silastic tube (1 cm length), and four silastic tubes were implanted s.c. into the interscapular space. These silastic tubes were changed every 3 months. Doses of $E_2$ and progesterone were determined based on the results of a preliminary experiment in which the effects of these steroids on the uterine weight were examined.

### *Treatment of mice*

The study consisted of three experiments. In experiment I, female mice treated with 3′-Me-DAB neonatally were divided into two groups. One group was ovariectomised at 1 month of age, and the other group was given a sham operation. Mice (10–22 mice) from each group were killed at 4, 6, 8, 10, 12 or 16 months of age, and the liver was removed promptly. In experiment II, female mice treated with 3′-Me-DAB were divided into five groups. Four groups were ovariectomised at 1 month of age, and one group was given a sham operation. The ovariectomised mice of three groups received s.c. implantation of $E_2$ pellets (10 mg), four silastic tubes, each containing about 12.5 mg of progesterone, or both $E_2$ pellets and four silastic tubes with progesterone at the time of the ovariectomy, and those of one group did not. $E_2$ pellets and silastic tubes with progesterone were changed every 4 and 3 months, respectively. Mice (10–20 mice) form each group were killed at 10 or 12 months of age, and the liver and uterus were removed promptly. In experiment III, female mice treated with 3′-Me-DAB were ovariectomised at 1 month of age. These mice were divided into three groups, and mice of each group received s.c. implantation of $E_2$ pellets (10 mg), or intrasplenic implantation of $E_2$ pellets (10 or 5 mg) at 6 months of age. Mice (22–34 mice) from each group were killed at 10 months of age, and the liver and uterus were removed promptly. At sacrifice of mice which had received intrasplenic implantation of $E_2$ pellets, adhesion of the spleen to the abdominal wall was examined carefully, and mice with the adhesion were excluded from the experiment.

*Pathological examination of adenomatous nodules*

The liver was fixed in Zamboni's solution and cut into 4-mm thick serial strips. A thin section of each strip was prepared and stained with hematoxylin and eosin, and all sections (12–15 sections/the liver) were examined for adenomatous nodules. An adenomatous nodule of hepatocellular origin in the liver was defined with reference to previous reports (Vesselinovitch *et al.*, 1978; Frith *et al.*, 1980; Lipsky *et al.*, 1981*a*) as described previously (Yamamoto *et al.*, 1991) as a mixture of eosinophilic, basophilic, vacuolated and foamy hepatocyte in various proportions that compressed the adjacent parenchyma, but did not contain a carcinomatous lesion with a trabecular structure. Number of adenomatous nodules ranged from one to three per the liver.

*Histochemical examination of glucose-6-phosphatase*

The right lobe of the liver was removed, and promptly frozen in liquid nitrogen. A frozen section of the widest area was cut at in 5–6 μm thickness on a cryostat and stained for glucose-6-phosphatase (G-6-Pase) by the method of Wachstein and Meisel (1957) and examined for G-6-Pase deficient foci. All adenomatous nodules were G-6-Pase deficient. Thus, G-6-Pase deficient foci (Figure 1) include both preneoplastic lesions and adenomatous nodules (Moore *et al.*, 1981; Lipsky *et al.*, 1981*b*; Hacker *et al.*, 1991). Number of G-6-Pase deficient foci ranged from one to six per a section of the widest area of the right lobe of the liver.

**Figure 1** G-6-Pase-deficient foci. Small arrows, preneoplastic G-6-Pase-deficient foci. Large arrow, an adenomatous nodule. Bar = 1 mm.

*Labelling index*

For examination of the labelling indices of adenomatous nodules, mice were given an i.p. injection of [methyl-$^3$H] thymidine (2 μCi $g^{-1}$ body wt; 86.4 Ci $mmol^{-1}$; New England Nuclear Corp., Boston, MA, USA), and were killed 4 h later. After fixation in Zamboni's solution, the liver was cut into 4 mm thick serial strips. Autoradiography of a thin section of each strip was carried out by exposure for 21 days as described previously (Terada *et al.*, 1989). The labelling index of adenomatous nodules with an average diameter of 3–5 mm was determined. The presence of more than five grains on the nucleus was considered to indicate positive labelling.

*Statistical analyses*

Statistical analyses were carried out by the Chi-square test or Student's *t*-test. A *P* value of less than 0.05 was regarded as significant.

### Results

Figure 2 shows the effect of ovariectomy of 1-month-old female mice treated with 3′-Me-DAB neonatally on the incidences of hepatocellular adenomatous nodules and G-6-Pase deficient foci. Adenomatous nodules and G-6-Pase deficient foci were first found in the liver of 16- and 12-month-old intact females, respectively. However, in the liver of ovariectomised females, adenomatous nodules and G-6-Pase deficient foci were first found at 8 and 6 months of age, respectively, and their incidences increased gradually thereafter. The incidences of adenomatous nodules and G-6-Pase deficient foci in ovariectomised females were significantly higher than those in intact females of the same age. Carcinoma was found only in one of 14 ovariectomised females at 16 months of age, and not in intact females of the

**Figure 2** Effects of ovariectomy on the incidences of adenomatous nodules and G-6-Pase-deficient foci. Female mice were treated with 3′-Me-DAB neonatally. One group of mice was ovariectomised at 1 month of age (■), and the other was not (□). Mice were killed at 4, 6, 8, 10, 12 or 16 months of age. Numbers above columns indicate numbers of mice examined [a]Significant difference from the value for intact females by the Chi-square test ($P<0.05$)

**Figure 3** Effects of estradiol-17$\beta$ and progesterone on the incidences of adenomatous nodules and G-6-Pase-deficient foci. Female mice were treated with 3′-Me-DAB neonatally. As indicated, groups of mice were ovariectomised at 1 month of age, with or without prompt implantation of $E_2$ pellets (10 mg), four silastic tubes (1 cm length) containing about 12.5 mg of progesterone (P), or both $E_2$ pellets and four silastic tubes with progesterone. Mice were killed at 10 or 12 months of age. Numbers above columns indicate numbers of mice examined. [a]Significant difference from the value for females ovariectomised at 1 month of age without implantation by the Chi-square test ($P<0.05$).

same age. The above-mentioned results suggest that ovarian hormones suppress the development of adenomatous nodules and G-6-Pase deficient foci, so we next examined the effects of oestrogen and progesterone on their development.

Figure 3 shows the effects of oestrogen and progesterone on the incidences of adenomatous nodules and G-6-Pase deficient foci in ovariectomised females at 10 or 12 months of age. Ovariectomy at 1 month of age resulted in marked increases in the incidences of adenomatous nodules and G-6-Pase deficient foci at 10 or 12 months of age, and s.c. implantation of $E_2$ pellets (10 mg) markedly decreased their incidences. On the other hand, s.c. implantation of four silastic tubes, each containing about 12.5 mg of progesterone did not decrease the incidences of adenomatous nodules and G-6-Pase deficient foci at 10 months of age, while s.c. implantation of both $E_2$ pellets and four silastic tubes containing progesterone reduced their incidences to the levels in the group with implantation of $E_2$ pellets only. Figure 4 shows the effects of oestrogen and progesterone on uterine weight. The uterine weight of ovariectomised females with implanted progesterone was significantly more than that of ovariectomised females without an implant, and implantation of progesterone plus $E_2$ pellets significantly decreased the uterine weight relative to that on implantation of $E_2$ pellets only.

The labelling indices (means ± SE) of adenomatous nodules of 3–5 mm diameter in intact females, females ovariectomised at 1 month of age, and females ovariectomised at 1 month of age with $E_2$ pellets implanted s.c. at 12 months of age were 1.24 ± 0.22% ($n = 5$), 3.10 ± 0.10% ($n = 9$), and 1.01 ± 0.14% ($n = 6$), respectively, at 15 months of age. The labelling index of adenomatous nodules in ovariectomised females was significantly ($P<0.05$) higher than that in intact females, and was reduced significantly ($P<0.05$) by implantation of $E_2$ pellets for 3 months.

Figure 5 shows the effects of s.c. and intrasplenic implantations of $E_2$ pellets at 6 months of age on the incidences of adenomatous nodules and G-6-Pase deficient foci at 10 months of age. The s.c. implantation of an $E_2$ pellet (10 mg) significantly reduced the incidences of both adenomatous nodules and G-6-Pase deficient foci, but the intrasplenic implantation of an $E_2$ pellet (10 or 5 mg) did not. The uterine weight of ovariectomised females with $E_2$ pellets implanted s.c. was comparable to that of females without ovariectomy (intact females), whereas the uterine weight of ovariectomised females with an $E_2$ pellet implanted into the spleen was as low as that of ovariectomised females without an implant (Figure 6). In the experiment shown in Figure 5, mice received the intrasplenic implantation of $E_2$ pellets were excluded from the experiment when adhesion of the spleen to the abdominal wall was found at sacrifice. The uterine weight of these mice with intrasplenic $E_2$ pellets (10 mg) was 116.8 ± 23.0 mg (mean ± SE for eight mice), ranging from 43.2 to 238.3 mg).

### Discussion

Carcinogen-induced preneoplastic and neoplastic lesions in rat and mice show alterations in activities of various enzymes including G-6-Pase activity (Bannasch, 1986). Hacker *et al.* (1991) reported that diethylnitrosamine-induced preneoplastic lesions in mice were G-6-Pase deficient, but adenomas showed increased or decreased G-6-Pase activity and carcinomas showed increased G-6-Pase activity. On the other hand,

**Figure 4** Effects of estradiol-17$\beta$ and progesterone on the uterine weight. Female mice were treated as described in the legend of Figure 3. Columns and bars represents means + SE and numbers indicate numbers of mice examined. [a]Significant difference from the value for ovariectomised females without implantation by the Student's *t*-test ($P<0.05$). [b]Significant difference from the value for ovariectromised females with $E_2$ pellets by the Student's *t*-test ($P<0.05$).

**Figure 5** Effects of estradiol-17$\beta$ implanted s.c. or into the spleen on the incidences of adenomatous nodules and G-6-Pase-deficient foci. Female mice were treated with 3′-Me-DAB neonatally. These females were ovariectomised at 1 month of age. At 6 months of age ovariectomised females received a $E_2$ pellet (10 mg) s.c. or a $E_2$ pellet (10 or 5 mg) in the spleen. All mice were killed at 10 months of age. Numbers above columns indicate numbers of mice examined. [a]Significant difference from the value for females ovariectomised at 1 month of age without implantation by the Chi-square test ($P<0.05$).

**Figure 6** Effects of estradiol-17β implanted s.c. or into the spleen on uterine weight. Female mice were treated as described in the legend of Figure 5. Columns and bars represent means + SE Numbers indicate numbers of mice examined. [a]Significant difference from the value for ovariectomised females without implantation by the Student's *t*-test ($P<0.05$).

Lipskey *et al.* (1981*b*; 1989) reported that safrole-induced or dieldrin and DDT-induced preneoplastic lesions, adenomas and carcinomas were all G-6-Pase deficient. In the present study, adenomatous nodules were G-6-Pase deficient, and were included in G-6-Pase deficient lesions. However, since G-6-Pase deficient lesions appeared earlier than adenomatous nodules both in intact and ovariectomised females treated with 3′-Me-DAB neonatally, G-6-Pase deficient lesions except adenomatous nodules seem to be preneoplastic lesions as suggested in hepatocellular tumourigenesis induced by the other carcinogens (Frith *et al.*, 1980; Lipsky *et al.*, 1981*a*; 1981*b*; 1989; Moore *et al.*, 1981; Vesselinovitch & Mihailovich, 1983). Our previous study (Yamamoto *et al.*, 1991) using both female and male mice treated with 3′-Me-DAB neonatally, showed that adenomatous nodules appeared earlier than carcinomas in males, and that the marked decrease in incidence of adenomatous nodules in females was accompanied by the marked decrease in incidence of carcinomas. Thus, it is also likely that carcinomas may arise from adenomatous nodules.

$E_2$ suppressed the development of both adenomatous nodules and G-6-Pase deficient foci. G-6-Pase deficient foci include both preneoplastic lesions and adenomatous nodules (Moore *et al.*, 1981; Lipsky *et al.*, 1981*a*; 1981*b*; 1989; Hacker *et al.*, 1991). Thus, this result indicates that $E_2$ suppresses the formation of preneoplastic lesions from initiated hepatocytes as well as the progression of preneoplastic lesions into adenomatous nodules. Furthermore, as $E_2$ decreased the labelling index of adenomatous nodules, it also seems to suppress growth of adenomatous nodules. These results are consistent with a report by Goldfarb and Pugh (1990) that ovariectomy accelerated the growth of hepatocellular neoplasms of mice induced by diethylnitrosamine.

Although there are no reports on the effects of $E_2$, the most potent natural oestrogen, on hepatocellular tumourigenesis in mice, there are several reports on the effects of synthetic oestrogens on hepatocellular tumourigenesis in mice and rats. Studies on the effects of synthetic oestrogens on hepatocellular tumourigenesis in mice have, however, given discrepant results (Lee *et al.*, 1989; Vesselinovitch & Mihailovich, 1982). Lee *et al.* (1989) reported that ethynyl estradiol suppressed the development of G-6-Pase deficient foci in various strains of mice induced by neonatal administration of diethylnitrosamine, whereas Vesselinovitch and Mihailovich (1982) reported that mestranol enhanced the development of mouse liver tumours induced by the same carcinogen. Furthermore, many studies in rats showed that treatment with synthetic oestrogens following carcinogen exposure enhanced the development of liver tumours (Metzler & Degen, 1987). Most synthetic oestrogens exert toxic effects on the rodent liver, which may contribute for their effects on the development of liver tumours. Therefore it seems necessary to use a natural oestrogen, estradiol-17β, to examine the effects of an ovarian hormone on hepatocellular tumourigenesis induced by various carcinogens in rats and mice.

The s.c. implantation of $E_2$ pellets suppressed the development of adenomatous nodules and G-6-Pase deficient foci, but the intrasplenic implantation of an $E_2$ pellet did not. As all the $E_2$ released from the intrasplenic pellet enters the portal vein, $E_2$ cannot exert actions on tissues other than the liver (Samuel & Eik-Nes, 1968). In contrast, $E_2$ released from s.c. pellets enters the systemic circulation, and can exert actions on extrahepatic tissues as well as the liver. Consistent with this, $E_2$ implanted into the spleen did not increase uterine weight, whereas $E_2$ implanted s.c. increased uterine weight to about that in intact females. In the present study, mice which had received the intrasplenic implantation of $E_2$ pellets were excluded from the experiment when adhesion of the spleen to the abdominal wall was found at sacrifice, since in these mice, a part of $E_2$ secreted from $E_2$ pellets enters the systemic circulation through veins of the abdominal wall. The uterine weights of some of these mice with $E_2$ pellets (10 mg) were comparable to those of mice which had received the s.c. implantation with $E_2$ pellets, suggesting that intrasplenic $E_2$ pellets could secrete $E_2$ as long as s.c. $E_2$ pellets. Thus, it is unlikely that no suppressive effects of the intrasplenic implantation of $E_2$ pellets is ascribed to the rapid absorption of $E_2$ from a pellet. Rather, the present results suggest that $E_2$ suppresses hepatocellular tumourigenesis in mice by its actions on tissue other than the liver. Since oestrogen modulates the secretion of pituitary hormones, especially prolactin (Toh, 1973; Meites, 1974), the pituitary gland may be involved in the suppressive effects of oestrogen on hepatocellular tumourigenesis. On the other hand, Goldfarb and Pugh (1990) have suggested that the promoting effect of ovariectomy on hepatocellular tumourigenesis in mice may be related to weight gain, as ovariectomy results in weight gain and as obesity, either strain specific or induced by gold thioglucose, is associated with a shortening of the latency in liver tumour development in mice (Waxler & Tabar, 1953; Gray *et al.*, 1960).

In this study we found that s.c. implantation of $E_2$ decreased the incidences of adenomatous nodules and G-6-Pase deficient foci, but that s.c. implantation of progesterone did not. However, progesterone implanted s.c. significantly increased the uterine weight of ovariectomised mice and inhibited the increase in uterine weight induced by $E_2$, indicating that when implanted s.c., it was an effective source of progesterone (Terada *et al.*, 1989). Thus, it is likely that progesterone does not affect hepatocellular tumourigenesis, and that only oestrogen secreted by the ovaries is responsible for the suppressive effect of the ovaries on hepatocellular tumourigenesis in mice.

We conclude from the present results that ovarian oestrogen suppresses hepatocellular tumourigenesis by actions on tissues other than the liver.

We thank Mr M. Yamamoto and T. Hayashiji for animal care, Mr M. Takatori for technical advice and Dr M. Tsuji for helpful comments on the manuscript.

### References

BANNASCH, P. (1986). Preneoplastic lesions as end points in carcinogenicity testing. I. Hepatic preneoplasia. *Carcinogenesis*, **7**, 689–695.

FRITH, G.H., BAETCKE, K.P., NELSON, C.J. & SCHIEFERSTEIN, G. (1980). Sequential morphogenesis of liver tumors in mice given benzidine dihydrochloride. *Europ. J. Cancer*, **16**, 1205–1216.

GOLDFARB, S. & PUGH, T.D. (1990). Ovariectomy accelerates the growth of microscopic hepatocellular neoplasms in the mouse: Possible association with whole body growth and fat desposition. *Cancer Res.*, **50**, 6779–6782.

GRAY, G.F., LIEBELT, R.A. & LIEBELT, A.G. (1960). The development of liver tumors in gold thioglucose-treated CBA mice. *Cancer Res.*, **20**, 1101–1104.

HACKER, H.J., MTIRO, H., BANNASCH, P. & VESSELIOVITCH, S.D. (1991). Histochemical profile of mouse hepatocellular adenomas and carcinomas induced by a single dose of diethylnitrosamine. *Cancer Res.*, **51**, 1952–1958.

KEMP, C.J., LEARY, C.N. & DRINKWATER, N.R. (1989). Promotion of murine hepatocarcinogenesis by testosterone is androgen receptor-dependent but not cell autonomous. *Proc. Natl Acad. Sci. USA*, **86**, 7505–7509.

KLEIN, M. (1959). Influence of low dose of 2-acetylaminofluorene on liver tumorigenesis in mice. *Proc. Soc. Exp. Biol. Med.*, **101**, 637–638.

KLEIN, M. & WEISBURGER, E.K. (1966). Carcinogenic effect of N-hydroxy-N-2-fluorenylacetamide, 2′ 4′-dimethylacetanilide, and 2′ 4′ 6′-trimethylacetanilide on liver in suckling mice. *Proc. Soc. Exp. Biol. Med.*, **122**, 111–114.

LEE, G.H., NAMURA, K. & KITAGAWA, T. (1989). Comparative study of diethylnitosamine-initiated two-stage heptocarcinogenesis in C3H, C57BL and BALB mice promoted by various hepatopromoters. *Carcinogenesis*, **10**, 2227–2230.

LIPSKY, M.M., HINTON, D.E., KLAUNIG, J.E. & TRUMP, B.F. (1981*a*). Biology of hepatocellular neoplasia in the mouse, I. Histogenesis of safrole-induced hepatocellular carcinoma. *J. Natl Cancer Inst.*, **67**, 365–376.

LIPSKY, M.M., HINTON, D.E., KLAUNIG, J.E., GOLDBLATT, P.J. & TRUMP, B.F. (1981*b*). Biology of hepatocellular neoplasia in the mouse. II. Sequential enzyme histochemical analysis of BALB/c mouse liver during safrole-induced carcinogenesis. *J. Natl Cancer Inst.*, **67**, 377–392.

LIPSKY, M.M. & TRUMP, B.F. (1989). Histogenesis of dieldrin and DDT-induced hepatocellular carcinoma in Balb/c mice. *J. Environ. Pathol. Toxicol. Oncol.*, **91**, 79–93.

MEITES, J. (1974). Relation of estrogen to prolactin secretion in animals and man. *Adv. Biosci.*, **4**, 195–208.

METZLER, M. & DEGEN, G.H. (1987). Sex hormones and neoplasia: liver tumors in rodents. *Arch. Toxicol Suppl.*, **10**, 251–263.

MOORE, M.R., DRINKWATER, N.R., MILLER, E.C., MILLER, J.A. & PITOT, H.C. (1981). Quantitative analysis of the time-dependent development of glucose-6-phosphatase-deficient foci in the liver of mice treated neonatally with diethylnitorosamine. *Cancer Res.*, **41**, 1585–1593.

RAO, K.V.N. & VESSELINOVITCH, S.D. (1973). Age- and sex-associated diethylnitrosamine dealkylation activity of the mouse liver and hepatocarcinogenesis. *Cancer Res.*, **33**, 1625–1627.

ROE, F.J.C., WARWICK, G.P., CARTER, R.L., PETO, R., ROSS, W.C.J., MITCHLEY, B.C.V. & BARRON, N.A. (1971). Liver and lung tumors in mice exposed at birth to 4-dimethylaminoazobenzene or its 2-methyl or 3′-methyl derivatives. *J. Natl Cancer Inst.*, **47**, 593–599.

SAMUEL, L.T. & EIK-NES, K.B. (1968). Metabolism of steroid hormones. In *Metabolic Pathways, Vol. 2: Third edition*, Greenberg, D.M. (ed.) pp. 169–220. Academic Press: New York and London.

TERADA, N., YAMAMOTO, R., TAKADA, T., MIYAKE, T., TERAKAWA, N., WAKIMOTO, H., TANIGUCHI, H., LI, W., KITAMURA, Y. & MATSUMOTO, K. (1989). Inhibitory effect of progesterone on cell death of mouse uterine epithelium. *J. Steroid Biochem.*, **33**, 1091–1096.

TOH, Y.C. (1973). Physiological and biochemical reviews of sex differences and carcinogenesis with particular reference to the liver. *Advan. Cancer Res.*, **18**, 155–209.

VESSELINOVITCH, S.D. & MIHAILOVICH, N. (1967). The effect of gonadectomy on the development of hepatomas induced by urethan. *Cancer Res.*, **27**, 1788–1791.

VESSELINOVITCH, S.D. (1969). The sex-dependent difference in the development of liver tumors in mice administered dimethylnitrosamine. *Cancer Res.*, **29**, 1024–1027.

VESSELINOVITCH, S.D., MIHAILOVICH, N., WAGAN, G.N., LOMBARD, L.S. & RAO, K.V.N. (1972). Alfatoxin $B_1$, a hepatocarcinogen in the infant mouse. *Cancer Res.*, **32**, 2289–2291.

VESSELINOVITCH, S.D., RAO, K.V.N., MIHAILOVICH, N., RICE, J.M. & LOMBARD, L.S. (1974). Development of broad spectrum of tumors by ethylnitrosourea in mice and the modifying role of age, sex and strain. *Cancer Res.*, **34**, 2530–2538.

VESSELINOVITCH, S.D., MIHAILOVICH, N. & RAO, K.V.N. (1978). Morphology and metastatic nature of induced hepatic nodular lesions in C57BL × C3H $F_1$ mice. *Cancer Res.*, **38**, 2003–2010.

VESSELINOVITCH, S.D., ITZE, L., MIHAILOVICH, N. & RAO, K.V.N. (1980). Modifying role of partial hepatectomy and gonadectomy in ethylnitrosourea-induced hepatocarcinogenesis. *Cancer Res.*, **40**, 1538–1542.

VESSELINOVITCH, S.D. & MIHAILOVICH, N. (1982). Modifying effects of synthetic steroids on the development of liver tumors induced by diethylnitrosamine. *Proc. Am. Assoc. Cancer Res.*, **23**, 91.

VESSELINOVITCH, S.D. & MIHAILOVICH, N. (1983). Kinetics of diethylnitrosamine hepatocarcinogenesis in the infant mouse. *Cancer Res.*, **43**, 4253–4259.

WACHSTEIN, M. & MEISEL, E. (1957). Histochemistry of hepatic phosphatases at a physiologic pH. *Am. J. Clin. Pathol.*, **27**, 13–23.

WAXLER, S.H. & TABAR, P. (1953). Appearance of hepatomas in obese C3H male mice. *Stanford Med. Bull.*, **11**, 272–273.

WEGHORST, C.M. & KLAUNIG, J.E. (1989). Phenobarbital promotion in diethylnitrosamine-initiated infant B6C3F1 mice: influence of gender. *Carcinogenesis*, **10**, 609–612.

YAMAMOTO, R., IISHI, H., TATSUTA, M., TSUJI, M. & TERADA, N. (1991). Roles of ovaries and testes in hepatocellular tumorigenesis induced in mice by 3′-methyl-4-dimethylaminoazobenzene. *Int. J. Cancer*, **49**, 83–88.